# Integrated bioinformatics analysis of differentially expressed genes and immune cell infiltration characteristics in Esophageal Squamous cell carcinoma

**DOI:** 10.1038/s41598-021-96274-y

**Published:** 2021-08-17

**Authors:** Zitong Feng, Jingge Qu, Xiao Liu, Jinghui Liang, Yongmeng Li, Jin Jiang, Huiying Zhang, Hui Tian

**Affiliations:** 1 Department of Thoracic Surgery, Qilu Hospital, Cheeloo College of Medicine, Shandong University, Jinan, 250012 Shandong China; 2 Department of Rheumatology and Clinical Immunology, Peking Union Medical College Hospital, Peking Union Medical College & Chinese Academy of Medical Sciences, Beijing, 100730 China; 3 Department of Pulmonary and Critical Care Medicine, Qilu Hospital, Cheeloo College of Medicine, Shandong University, Jinan, 250012 Shandong China; 4 Laboratory of Basic Medical Sciences, Qilu Hospital, Cheeloo College of Medicine, Shandong University, Jinan, 250012 Shandong China

**Keywords:** Cancer, Computational biology and bioinformatics, Biomarkers, Oncology

## Abstract

Esophageal squamous cell carcinoma (ESCC) is a life-threatening thoracic tumor with a poor prognosis. The role of molecular alterations and the immune microenvironment in ESCC development has not been fully elucidated. The present study aimed to elucidate key candidate genes and immune cell infiltration characteristics in ESCC by integrated bioinformatics analysis. Nine gene expression datasets from the Gene Expression Omnibus (GEO) database were analysed to identify robust differentially expressed genes (DEGs) using the robust rank aggregation (RRA) algorithm. Functional enrichment analyses showed that the 152 robust DEGs are involved in multiple processes in the tumor microenvironment (TME). Immune cell infiltration analysis based on the 9 normalized GEO microarray datasets was conducted with the CIBERSORT algorithm. The changes in macrophages between ESCC and normal tissues were particularly obvious. In ESCC tissues, M0 and M1 macrophages were increased dramatically, while M2 macrophages were decreased. A robust DEG-based protein–protein interaction (PPI) network was used for hub gene selection with the CytoHubba plugin in Cytoscape. Nine hub genes (CDA, CXCL1, IGFBP3, MMP3, MMP11, PLAU, SERPINE1, SPP1 and VCAN) had high diagnostic efficiency for ESCC according to receiver operating characteristic (ROC) curve analysis. The expression of all hub genes except MMP3 and PLAU was significantly related to macrophage infiltration. Univariate and multivariate regression analyses showed that a 7-gene signature constructed from the robust DEGs was useful for predicting ESCC prognosis. Our results might facilitate the exploration of potential targeted TME therapies and prognostic evaluation in ESCC.

## Introduction

Esophageal cancer is the seventh most common cancer worldwide, with an estimated 572,034 new cases and 508,585 deaths occurring in 2018^1^. Esophageal squamous cell carcinoma (ESCC) accounts for approximately 90% of new incident esophageal cancers each year^2^. Due to its inconspicuous symptoms and inadequate endoscopic screening, esophageal cancer is often diagnosed at an advanced stage, and the 5-year overall survival (OS) rate ranges from 12 to 20%^3^. Recently, minimally invasive esophagectomy (MIE), neoadjuvant chemoradiotherapy, targeted therapy and immunotherapy have emerged. These multimodal therapeutic advances have shown promising results, but a substantial fraction of patients fail to benefit, and the massive burden in new ESCC cases may continue to increase given population growth and ageing. Therefore, a much more comprehensive analysis of the molecular mechanisms and underlying immune microenvironment is needed to further progress in combating ESCC.

Over the past decades, nucleic acid and protein sequence and structure data have increased exponentially. Bioinformatics analyses using in silico techniques is an important component of tumor research in aspects such as cancer-related gene discovery, clinical diagnosis, new drug molecular target discovery, and innovative drug design. Recently, novel checkpoint kinase 1 (CHK1) inhibitor determinants and specific natural inhibitors of cyclin-dependent kinases (CDKs) were detected based on an integrated in silico protocol^4,5^. Through many computational methodologies, several molecules could be developed as anticancer drugs with targeting effects on mitotic kinases^6,7^. Investigations of ras-related C3 botulinum toxin substrate 1 (RAC1) mutations through in silico approaches revealed that the pathogenic point mutation P29S would facilitate the design of tumour-targeting treatments^8^. In addition, comprehensive analysis of well-established databases indicated that single-nucleotide polymorphisms (SNPs) in microRNA binding sites of centrosomal protein (CEP) genes could serve as potential therapeutic targets in centrosome-associated cancers^9^.

A group of ESCC-related candidate genes were discovered in previous studies by analyses with public databases and high-throughput platforms such as Gene Expression Omnibus (GEO) and The Cancer Genome Atlas (TCGA). Zhang et al*.*^10^ identified 345 differentially expressed genes (DEGs) in ESCC based on three GEO datasets, and five hub genes have use as potential prognostic biomarkers. Based on TCGA database analysis, a prognostic model constructed from 9 immune-related genes classified patients into two groups with different outcomes, and M0 and M2 macrophages were significantly enriched in the high-risk group^11^. Using 5 GEO datasets, Karagoz et al.^12^ analysed transcriptional regulatory networks, reporter metabolic features and molecular pathways mediating ESCC development. However, the dataset and sample sizes used for these ESCC integrated omics studies were relatively small. The robust rank aggregation (RRA) algorithm can reduce outliers and inconsistent results caused by different platforms and analysis methods, but few reports of integrating more datasets in ESCC research using the RRA algorithm are available.

This study aimed to identify robust DEGs and characterize the immune cell infiltration distribution in ESCC from as many datasets as possible. In addition, a prognostic model for ESCC based on the robust DEGs was established. Enrichment analysis and immune infiltration analysis of robust DEGs would improve the understanding of the molecular mechanisms of tumorigenesis and facilitate the development of new therapeutic strategies in ESCC.

## Results

### Identification of DEGs and robust DEGs

**Figure 1:**
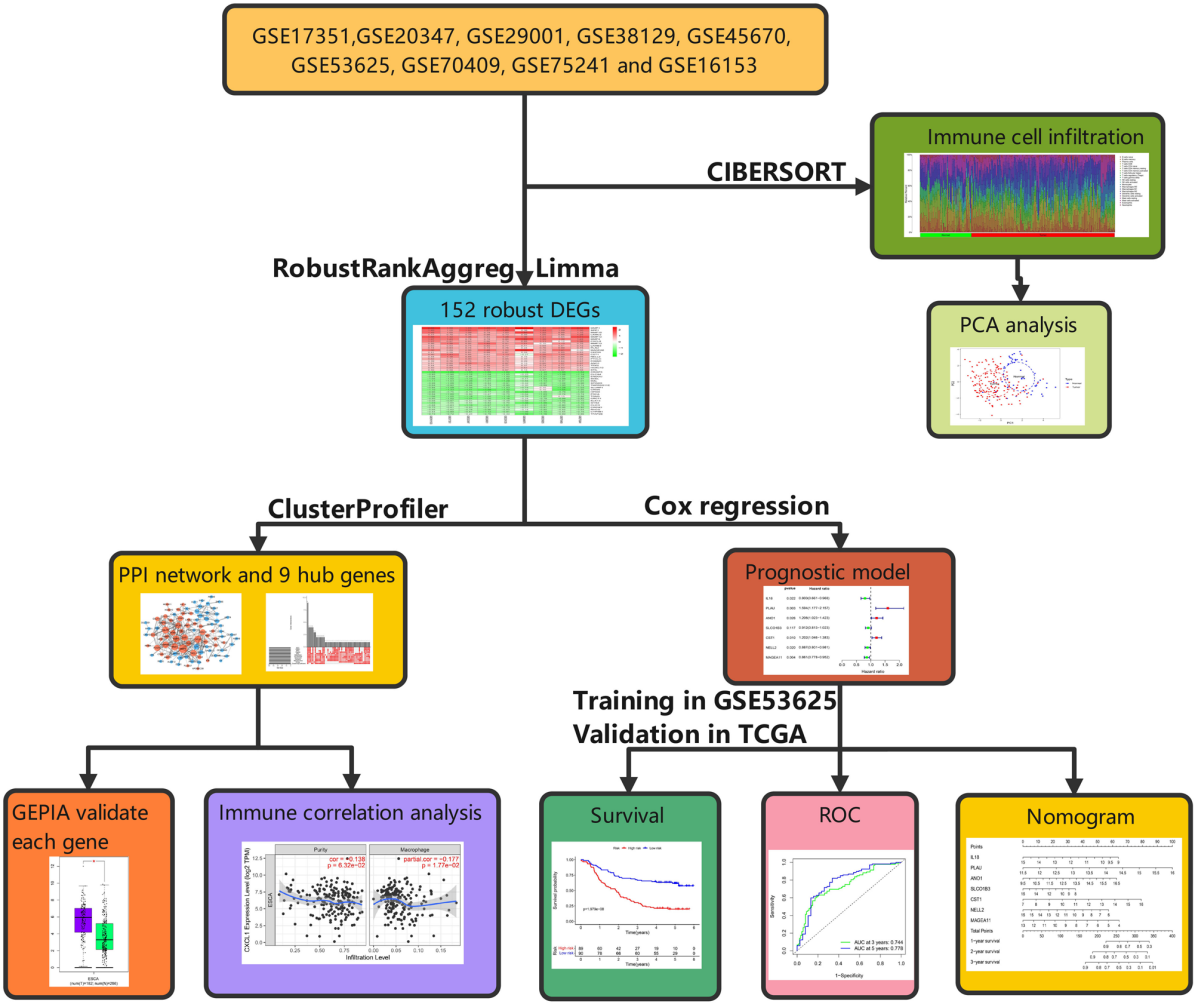
Flowchart of the integrated computational strategy used to analyse differentially expressed genes in ESCC.

**Figure 2:**
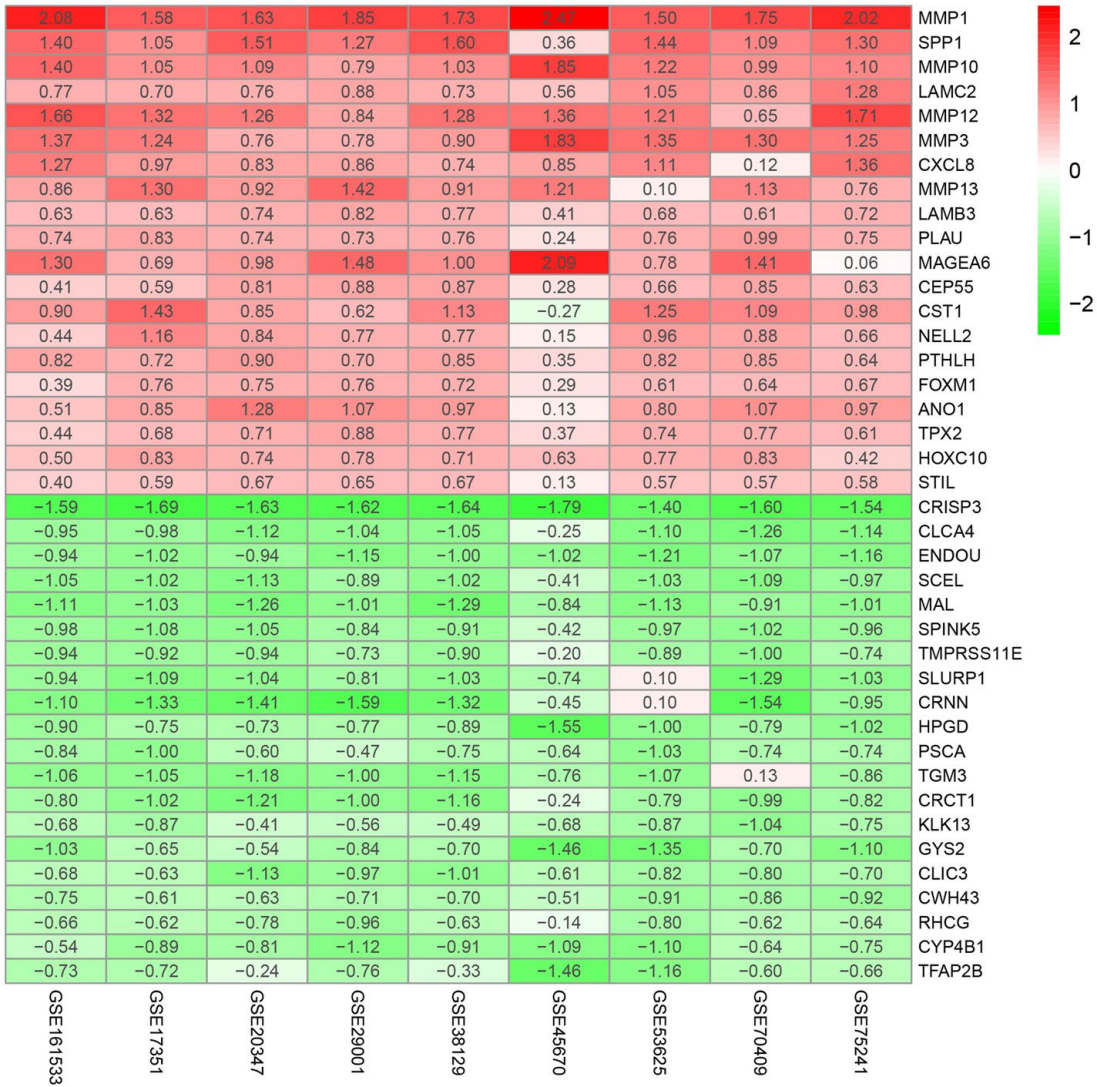
Top 20 robust DEGs in ESCC vs. normal tissues. Heatmap of the top 20 upregulated and downregulated robust DEGs identified by the RRA algorithm. Red indicates robust DEGs with high expression, while green indicates robust DEGs with low expression. The heatmap was drawn using R software (version 3.6.3, https://www.r-project.org/). DEGs, differentially expressed genes; RRA, robust rank aggregation.

In the present study, we conducted a systematic analysis of the biological characteristics of DEGs from nine GEO datasets (Table 1). The overall study design is illustrated in Fig. 1. A total of 665 tissue samples, including 343 ESCC and 322 normal tissue samples, were analysed. According to the cutoff criteria of |log2 fold change (FC)|) > 2 and adjusted *P* < 0.05, 226 DEGs in GSE17351, 219 DEGs in GSE20347, 389 DEGs in GSE29001, 108 DEGs in GSE38129, 692 DEGs in GSE45670, 686 DEGs in GSE53625, 387 DEGs in GSE70409, 223 DEGs in GSE75241 and 147 DEGs in GSE161533 were identified. Among the DEGs in these respective datasets, 110, 56, 168, 38, 249, 204, 115, 124 and 57 genes were upregulated, while 116, 163, 221, 70, 443, 482, 272, 99 and 90 genes were downregulated. To visualize the distributions of the DEGs, volcano plots (Supplementary Fig. S1) and heat maps (Supplementary Fig. S2) were drawn. The RRA algorithm precluded the substantial heterogeneity and the error of each experiment caused by the different technological platforms and challenging statistical methods. We ranked the DEGs according to their log2FC values. The higher a gene ranked in all the datasets, the greater was the likelihood that it was a DEG. According to analysis with false discovery rate (FDR) < 0.05, 152 robust DEGs—54 upregulated and 98 downregulated—were identified (Supplementary Table S1). The top 20 upregulated and downregulated robust DEGs are shown in a heat map (Fig. 2).

**Table 1:** Basic information of the 9 GEO microarray datasets.

Datasets	Year	Country	Tumor/Normal	Follow-up	Platform	Number of rows
GSE17351	2009	USA	5/5	No	GPL570	54,675
GSE20347	2010	USA	17/17	No	GPL571	22,277
GSE29001	2011	USA	21/24	No	GPL571	22,277
GSE38129	2012	USA	30/30	No	GPL571	22,277
GSE45670	2013	China	28/10	No	GPL570	54,675
GSE53625	2013	China	179/179	Yes	GPL18109	71,584
GSE70409	2013	China	17/17	No	GPL13287	29,187
GSE75241	2015	Brazil	15/15	No	GPL5175	316,919
GSE161533	2020	China	28/28	No	GPL570	54,675

### Gene ontology (GO) and Kyoto Encyclopedia of Genes and Genomes (KEGG) enrichment analyses of the robust DEGs

**Figure 3:**
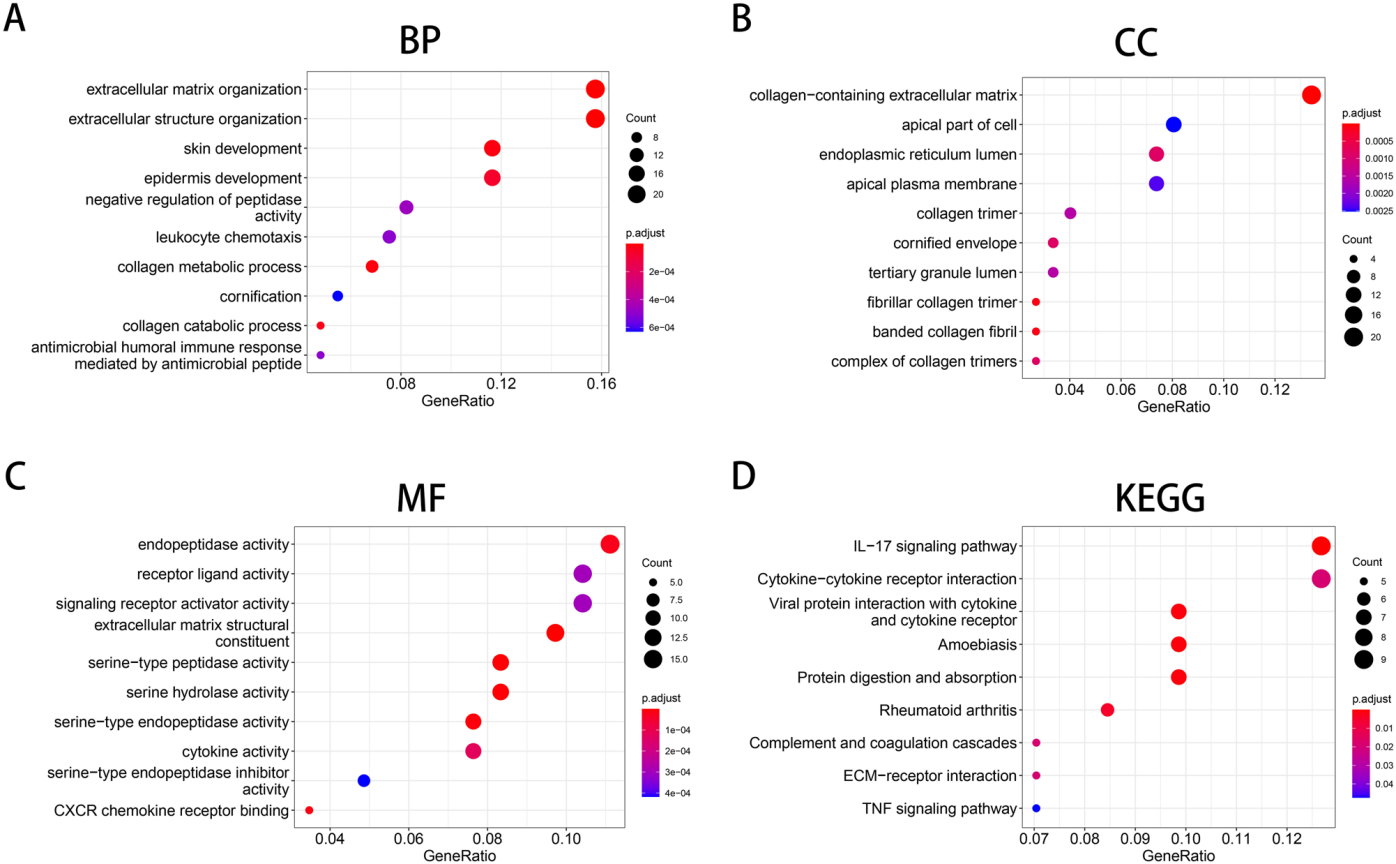
GO and KEGG pathways enriched with the robust DEGs in ESCC. (**A**) Biological process terms enriched with the robust DEGs. (**B**) Cellular component terms enriched with the robust DEGs. (**C**) Molecular function terms enriched with the robust DEGs. (**D**) KEGG analysis of the robust DEGs. The respective pathway involved in ESCC was identified by using the KEGG pathway database (https://www.kegg.jp/kegg/pathway.html). GO, Gene Ontology; KEGG, Kyoto Encyclopedia of Genes and Genomes.

To explore the biological classification of the 152 robust DEGs in ESCC, we performed GO and KEGG pathway enrichment analyses. Many biological functions enriched with the DEGs were associated with the tumor microenvironment (TME) and growth of cancer cells. GO enrichment analysis in the biological process (BP) category suggested that the robust DEGs were enriched in “extracellular matrix organization”, “extracellular structure organization” and “leukocyte chemotaxis” (Fig. 3A). In the cellular component (CC) category, the robust DEGs were enriched in “collagen-containing extracellular matrix”, “apical part of cell” and “endoplasmic reticulum lumen” (Fig. 3B). In the molecular function (MF) category, the robust DEGs were involved in “receptor ligand activity”, “signaling receptor activator activity”, “extracellular matrix structural”, “cytokine activity” and “CXCR chemokine receptor binding” (Fig. 3C). KEGG pathway analysis indicated that the robust DEGs were related to the following pathways: “IL-17 signaling pathway”, “cytokine-cytokine receptor interaction”, “ECM − receptor interaction” and “TNF signaling pathway” (Fig. 3D). The above results suggested that the abnormal expression of the robust DEGs may mediate tumor progression and TME remodelling.

### Characteristics of immune cell infiltration

**Figure 4:**
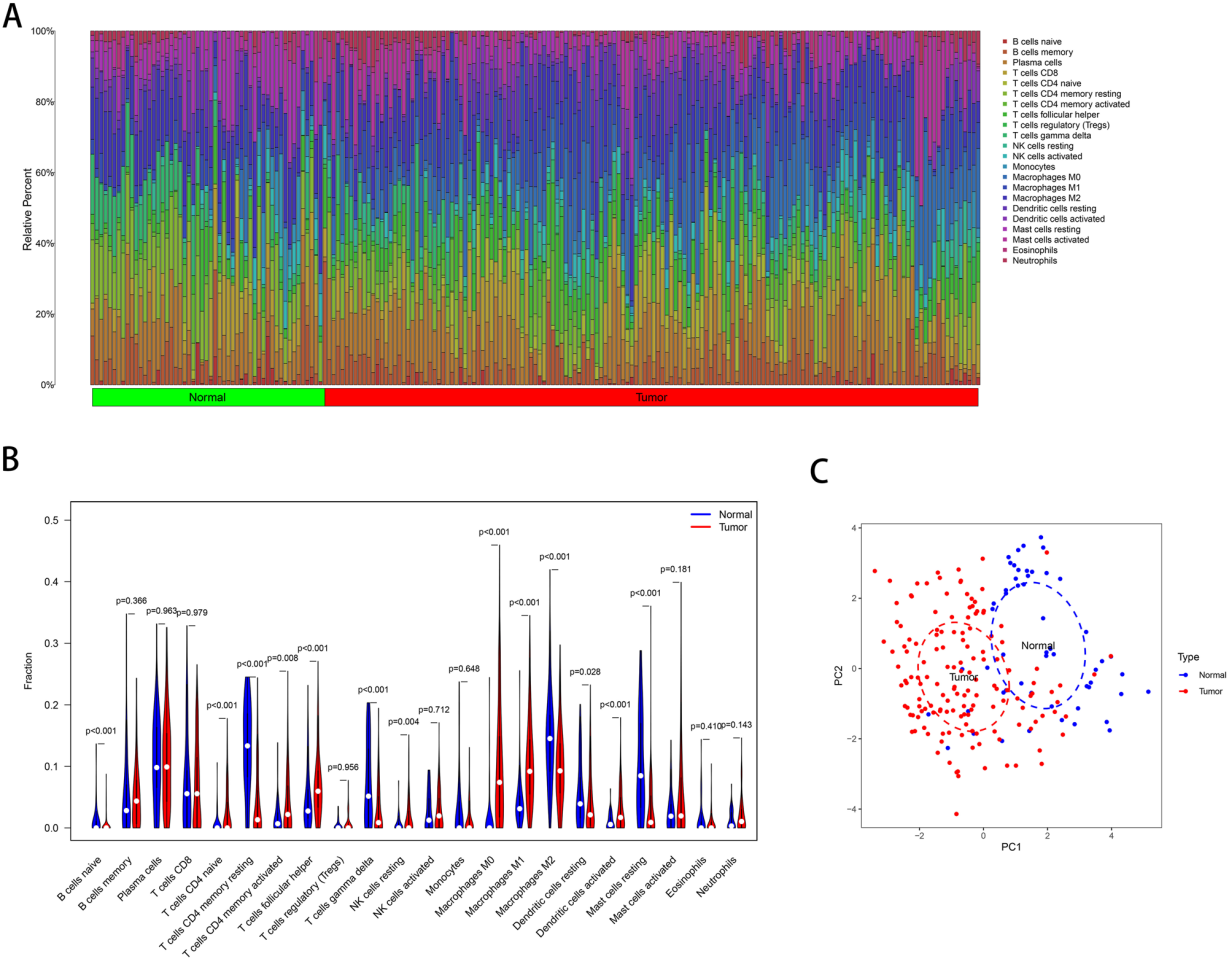
Characteristics of infiltrating immune cells. (**A**) Proportions of 22 immune cell subpopulations in ESCC and normal tissues. (**B**) Violin plot showing the immune cells with differential infiltration (*P* < 0.05). (**C**) PCA showed that 22 types of immune cells could roughly distinguish between ESCC and normal tissues.

Immune system cells infiltrating the TME are accepted to be generic constituents of tumors ^13^. The CIBERSORT algorithm was used to analyse immune cell infiltration in all 665 samples from the 9 GEO normalized expression matrices. The immune infiltration results were filtered with *P* < 0.05 as the criterion, and the proportions of 22 immune cells in 149 ESCC samples and 54 normal tissue samples were obtained (Fig. 4A). The heat map (Supplementary Fig. S3) and violin plot (Fig. 4B) offer further visualization of the differences in the immune cell distribution between ESCC and normal samples. Seven types of immune cells [naïve CD4^+^ T cells, activated memory CD4^+^ T cells, follicular helper T cells, resting natural killer (NK) cells, M0 macrophages, M1 macrophages and activated dendritic cells] were more abundant in ESCC tissues than in normal tissues, whereas 6 types of immune cells (naïve B cells, resting memory CD4^+^ T cells, gamma delta T cells, M2 macrophages, resting dendritic cells and resting mast cells) were more abundant in normal tissues. The changes in macrophages were particularly pronounced in ESCC tissues. As demonstrated by principal component analysis (PCA) (Fig. 4C), ESCC and normal samples could be roughly distinguished using the 22 immune cell types.

### Protein–protein interaction (PPI) network construction and identification of hub genes

**Figure 5:**
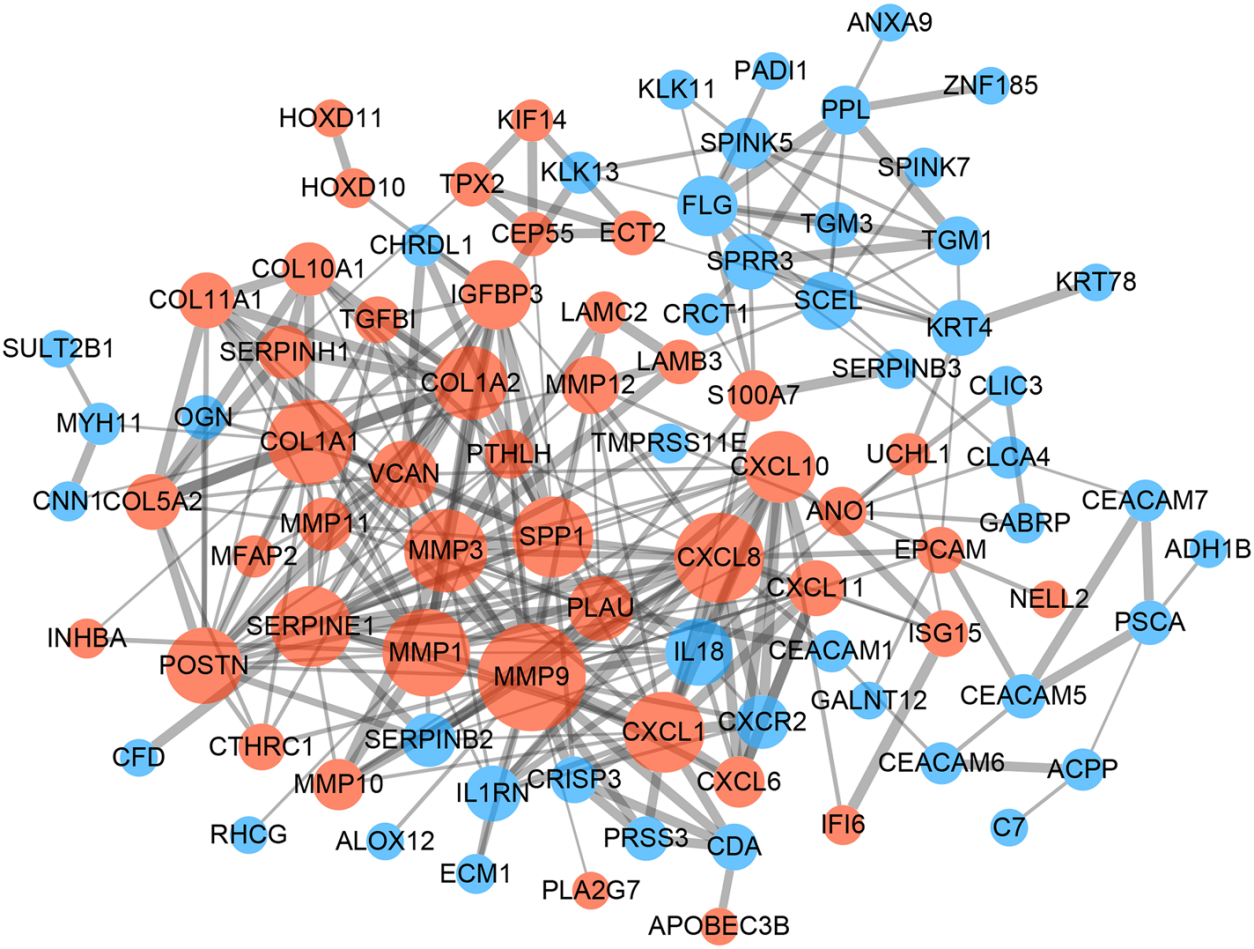
PPI network construction and hub gene identification. The PPI network consisted of 91 nodes and 304 edges. The red nodes indicate the upregulated genes, and the blue nodes indicate the downregulated DEGs. The node size represents the connectivity degree. The edge thickness represents the combined score. The network was visualized with Cytoscape software (Version 3.72, http://www.cytoscape.org/).

To further study the interaction of the 152 robust DEGs, we constructed a PPI network using the Search Tool for the Retrieval of Interacting Genes (STRING) database with a combined score > 0.4 as the cutoff criterion. As shown in Fig. 5, the PPI network including 45 upregulated genes and 46 downregulated genes contained 91 nodes and 304 edges. Subsequently, the cytoHubba plugin was used to calculate the scores of topological algorithms in each node. The genes with the 50 highest scores calculated by each of the 12 algorithms were intersected to identify the hub genes (Supplementary Fig. S4). The nine identified hub genes were cytidine deaminase (CDA), chemokine ligand 1 (CXCL1), insulin-like growth factor binding protein 3 (IGFBP3), matrix metallopeptidase 3 (MMP3), matrix metallopeptidase 11 (MMP11), plasminogen activator urokinase (PLAU, also named uPA), serpin peptidase inhibitor member 1 (SERPINE1), secreted phosphoprotein 1 (SPP1) and versican (VCAN).

### Validation of hub genes

**Figure 6:**
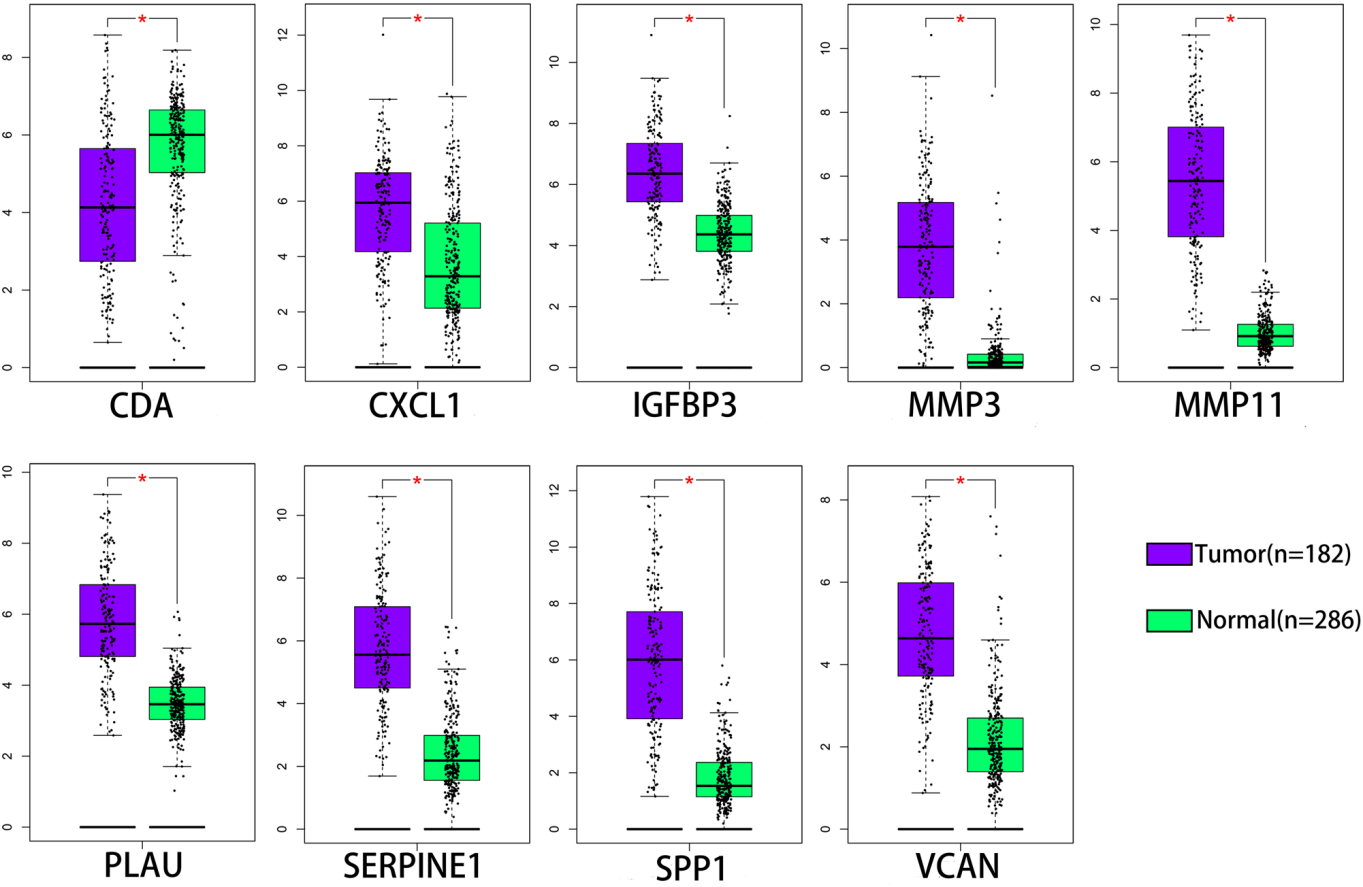
Expression of hub genes in ESCA tissues and normal tissues from the GEPIA database. ESCA, Esophageal carcinoma; GEPIA, Gene Expression Profiling Interactive Analysis. *P < 0.01 was considered statistically significant.

**Figure 7:**
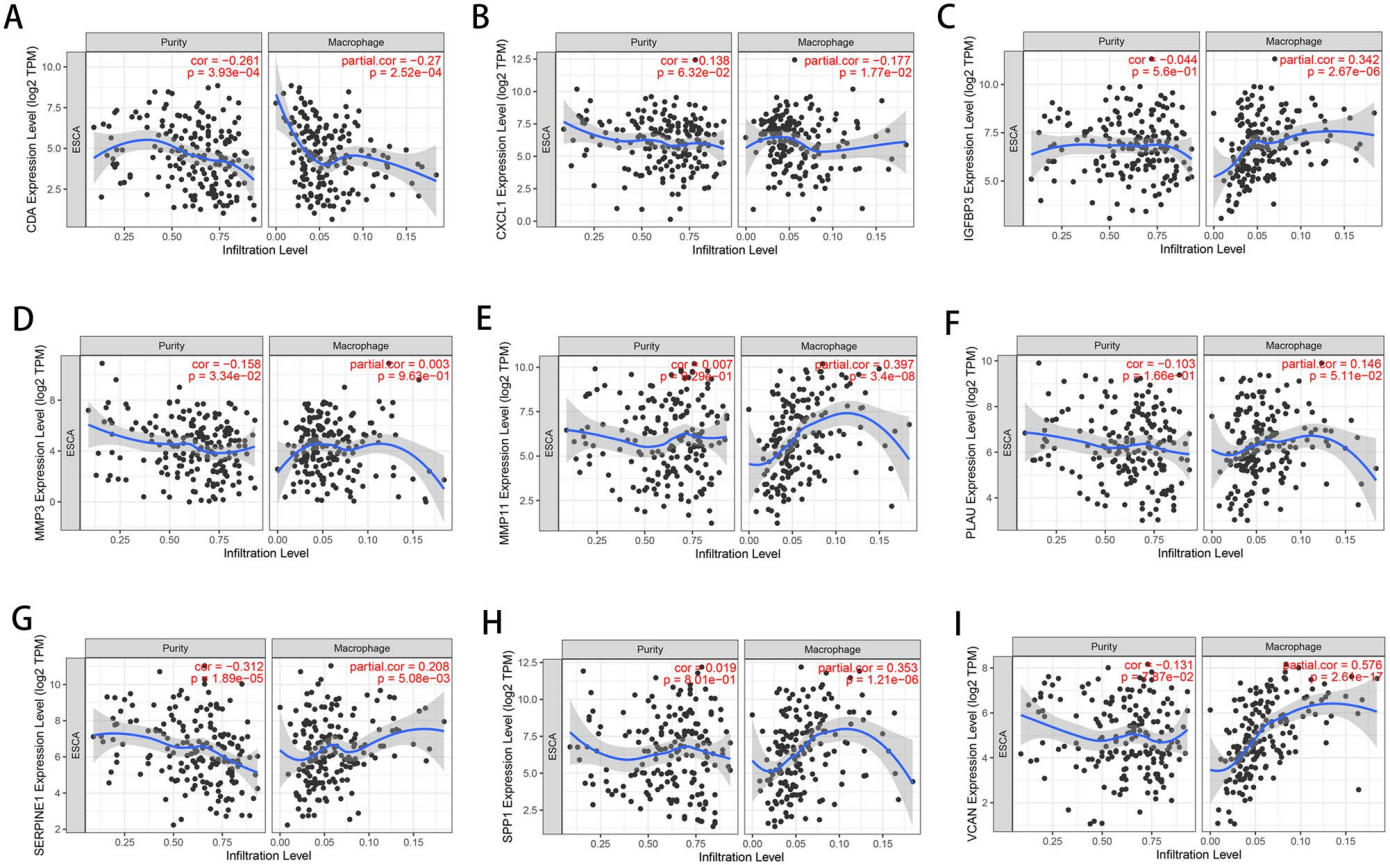
Correlations between hub genes and immune infiltration levels validated with the TIMER database. Correlation of the (**A**) CDA, (**B**) CXCL1, (**C**) IGFBP3, (**D**) MMP3, (**E**) MMP11, (**F**) PLAU, (**G**) SERPINE1, (**H**) SPP1 and (**I**) VCAN expression levels with the macrophage infiltration level, as well as the purity of tumor cells. TIMER, Tumor Immune Estimation Resource.

The mRNA expression of the 9 hub genes was validated using the Gene Expression Profiling Interactive Analysis (GEPIA) database. Consistent with the results of the GEO analysis, the mRNA expression of CXCL1, IFGFBP3, MMP3, MMP11, PLAU, SERPINE1, SPP1 and VCAN was markedly upregulated but the mRNA expression of CDA was markedly downregulated in esophageal carcinoma tissues (*P* < 0.01) (Fig. 6). Receiver operating characteristic (ROC) curves were generated to verify the diagnostic performance of these hub genes based on the GSE53625 database. The area under the curve (AUC) values of CDA, CXCL1, IGFBP3, MMP3, MMP11, PLAU, SERPINE1, SPP1 and VCAN were 0.8816, 0.8303, 0.9627, 0.9462, 0.9975, 0.9822, 0.9344, 0.9890 and 0.9454, respectively (Supplementary Fig. S5). The Tumor Immune Estimation Resource (TIMER) database was used to assess correlations between the mRNA expression levels of hub genes and the immune infiltration level (Fig. 7A-I). Our results showed that the expression levels of CDA (Cor =  − 0.27) and CXCL1 (Cor =  − 0.177) were negatively associated with the macrophage infiltration level. In contrast, those of IGFBP3 (Cor = 0.342), MMP11 (Cor = 0.397), PLAU (Cor = 0.146), SERPINE1 (Cor = 0.208), SPP1 (Cor = 0.353) and VCAN (Cor = 0.576) were positively associated with the macrophage infiltration level.

### Construction and verification of the prognostic model

**Figure 8:**
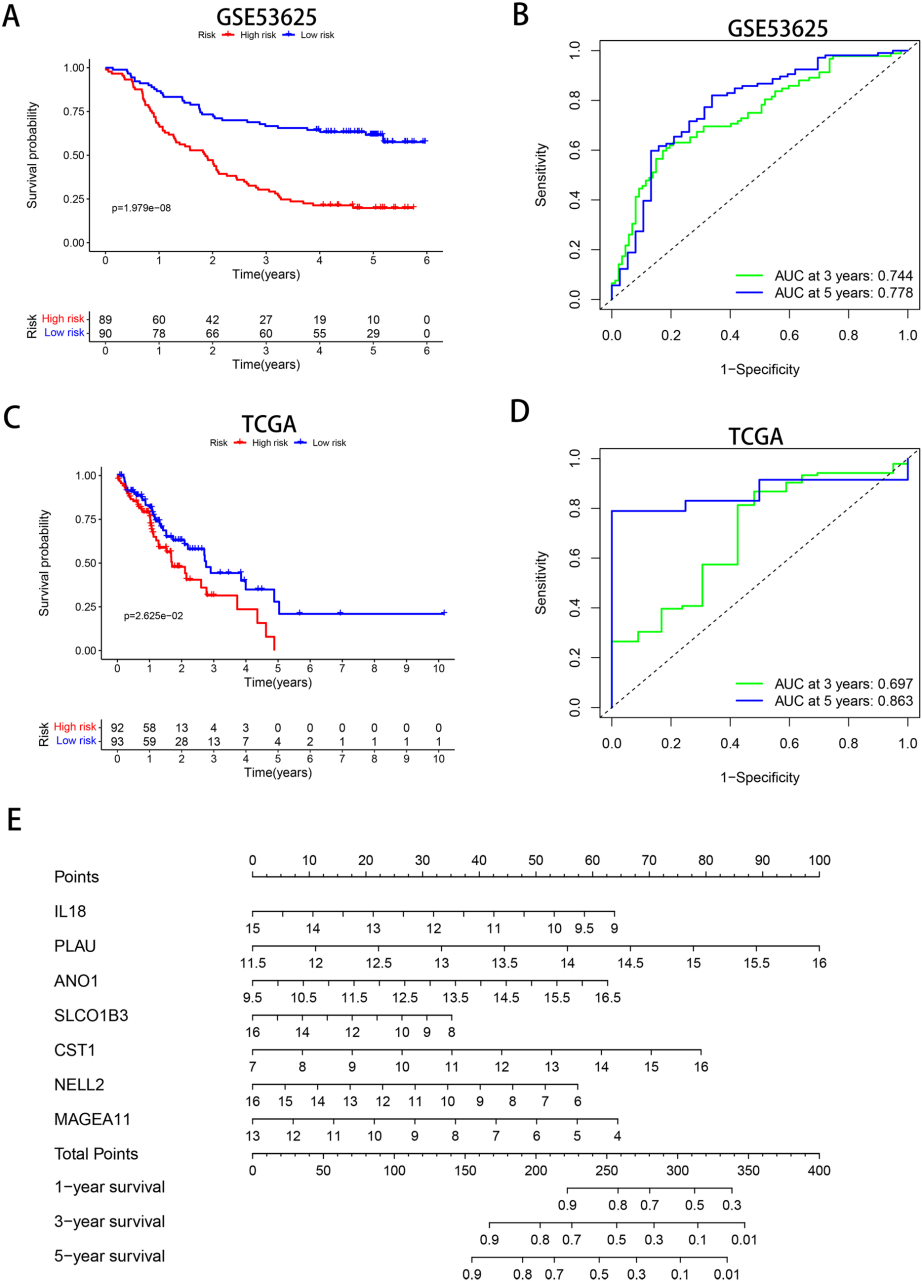
Analysis of the prognostic risk score model containing 7 prognostic genes in ESCC patients. (**A, C**) Kaplan–Meier survival analysis in the GSE53625 and TCGA cohorts (with patients grouped by the median risk score). (**B, D**) ROC curves for predicting 3- and 5-year OS based on the risk score in the GSE53625 and TCGA cohorts. (**E**) Nomogram for predicting the 1-, 3- and 5-year OS of ESCC patients in the GSE53625 cohort. ROC, receiver operating characteristic.

To investigate the prognostic significance of the 152 robust DEGs, 17 survival-related genes (*P* < 0.05) were identified by univariate Cox regression analysis in the GSE53625 dataset (Table 2). After selecting the most suitable combination of candidate genes by multiple stepwise Cox regression, seven genes—interleukin 18 (IL18), PLAU, anoctamin 1 (ANO1), solute carrier organic anion transporter family member 1B3 (SLCO1B3), cystatin SN (CST1), neural EGFL like 2 (NELL2) and melanoma antigen family A11 (MAGEA11), were used to construct a prognostic model (Table 3). The risk score of each patient was calculated according to the following formula: (-0.2232 × ExpIL18) + (0.4659 × ExpPLAU) + (0.1876 × ExpANO1) + (-0.0921 × ExpSLCO1B3) + (0.1844 × ExpCST1) + (-0.1203 × ExpNELL2) + (-0.1501 × ExpMAGEA11). As shown in Supplementary Fig.  S6, the expression heat map of the 7 prognostic genes was generated. To validate the risk model constructed with the 179 patients in GSE53625, we selected 185 patients in TCGA as the validation cohort. The patients in the two cohorts were divided into the low-risk and high-risk groups according to the median risk score. Kaplan–Meier survival analysis demonstrated that in both cohorts, the prognosis of the low-risk group was significantly better than that of the high-risk group (*P* < 0.05) (Fig. 8A,C). In addition, time-dependent ROC curve analysis revealed that the AUC values of the risk score in the training cohort were 0.744 and 0.778 for predicting 3- and 5-year overall survival (OS), respectively (Fig. 8B). In the validation cohort, the AUC values were 0.697 and 0.863 for predicting 3- and 5-year OS, respectively (Fig. 8D). These results indicated that the risk model constructed with the robust DEGs achieved good accuracy for evaluating patient prognosis.

**Table 2:** Univariate Cox regression analysis of the 17 genes.

Gene	HR	Lower 95%CI	Upper 95%CI	P
MYH11	1.214316	1.016687	1.450361	0.032148
CRCT1	0.81209	0.692012	0.953004	0.010785
IL18	0.792458	0.663518	0.946454	0.010248
CNN1	1.206791	1.01106	1.440414	0.037362
SERPINH1	1.527643	1.064343	2.192614	0.021552
SERPINB2	0.901575	0.817279	0.994566	0.03857
PLAU	1.414795	1.056845	1.893982	0.019729
SULT2B1	0.832297	0.735726	0.941542	0.003532
TMPRSS11E	0.840419	0.736972	0.958387	0.009481
KLK11	0.862082	0.76162	0.975795	0.018897
ANO1	1.265394	1.066067	1.50199	0.007113
SLCO1B3	0.863263	0.776586	0.959614	0.006458
CST1	1.205793	1.055109	1.377997	0.006004
NELL2	0.86076	0.779304	0.95073	0.003116
MAGEA6	0.91218	0.837226	0.993845	0.035634
MAGEA4	0.920913	0.859059	0.98722	0.020204
COL11A1	0.754961	0.571866	0.996678	0.047318

*HR* hazard ratio, *CI* confidence interval.

**Table 3:** Multivariate Cox regression analysis of the 7-gene signature.

Gene	Coef	HR	Lower 95%CI	Upper 95%CI	P
IL18	− 0.22318	0.799971	0.660934	0.968256	0.021956
PLAU	0.465937	1.593507	1.177179	2.157075	0.002563
ANO1	0.187636	1.206394	1.022512	1.423344	0.026161
SLCO1B3	− 0.09211	0.912006	0.812737	1.023399	0.117213
CST1	0.184351	1.202437	1.045682	1.382691	0.009689
NELL2	− 0.12029	0.886663	0.801369	0.981035	0.019753
MAGEA11	− 0.15012	0.860602	0.777913	0.95208	0.003583

*HR* hazard ratio, *CI* confidence interval.

### Verification of the independence of the prognostic risk model and construction of a prognostic nomogram

Cox regression analysis was carried out on the GSE53625 dataset to demonstrate whether the risk score is a valuable independent prognostic indicator. Univariate Cox regression analysis showed that pathologic N stage (*P* < 0.001), clinical stage (*P* < 0.001) and risk score (*P* < 0.001) were significantly correlated with OS. Multivariate Cox regression analysis showed that the risk score (*P* < 0.001) could serve as an independent prognostic factor to predict the OS of patients with ESCC (Table 4). To better predict the prognosis of patients with ESCC at 1, 3, and 5 years after esophagectomy, we integrated the 7-gene signature to establish a nomogram (Fig. 8E). A higher total number of points indicates a lower OS rate.

**Table 4:** Univariate and multivariate independent prognostic analyses of ESCC clinical characteristics based on the prediction model.

Variables	Univariate analysis		Multivariate analysis	
	Hazard ratio (95% CI)	P	Hazard ratio (95% CI)	P
Age	1.030 (1.008 − 1.053)	0.009	1.024 (1.000 − 1.048)	0.049
Gender	0.783 (0.489 − 1.252)	0.307	0.805 (0.495 − 1.311)	0.384
Grade	1.352 (1.002 − 1.823)	0.048	1.194 (0.892 − 1.598)	0.234
T	1.187 (0.910 − 1.549)	0.205	0.935 (0.655 − 1.334)	0.711
N	1.438 (1.181 − 1.751)	< 0.001	1.084 (0.794 − 1.481)	0.611
Stage	1.994 (1.398 − 2.846)	< 0.001	1.661 (0.910 − 3.031)	0.099
riskScore	1.728 (1.474 − 2.025)	< 0.001	1.603 (1.364 − 1.883)	< 0.001

## Discussion

Esophageal cancer is one of the most common cancers and exhibits high mortality worldwide due to its late diagnosis and lack of efficient treatment. Almost 50% of all esophageal cancer cases occur in China, and ESCC is the most dominant subtype^14^. A sophisticated understanding of the molecular mechanisms involved in ESCC progression and TME remodelling will facilitate the exploration of potential targets for its diagnosis and treatment.

In our study, we identified 152 robust DEGs—54 upregulated and 98 downregulated—by analysis of 9 gene expression microarray datasets. Interestingly, functional enrichment analysis demonstrated that the robust DEGs were significantly associated with many TME-related processes, such as “extracellular matrix organization”, “collagen-containing extracellular matrix”, “ECM − receptor interaction”, “CXCR chemokine receptor binding”, “IL-17 signaling pathway” and “TNF signaling pathway”. The TME is a complex environment in which tumor cells survive and is composed mainly of surrounding blood vessels, extracellular matrix (ECM), stromal cells, fibroblasts, immune cells and signalling molecules^15^. Dysregulation of ECM dynamics promotes tumor progression and tumor microenvironment formation^16^. Cancer hallmarks are affected by biophysical and biochemical signals from tumor-associated ECM. The mechanical properties and configuration of the ECM have been demonstrated to play important roles in sustaining proliferation, evading growth suppression, resisting cell death, enabling replicative immortality, inducing angiogenesis, activating invasion, avoiding immune destruction, deregulating cellular energetics, and facilitating genomic instability and tumor-promoting chronic inflammation^17^. Numerous interactions between esophageal cancer cells and the ECM seem to be intricate^18^. Many studies have demonstrated that different ECM molecules play a regulatory role in the development and metastasis of ESCC^19–21^. Regulation of chemokine and chemokine receptor (CXCR) signalling can remodel the immune phenotype in the TME. For example, the CXCR1/2 axis promotes the recruitment of immunosuppressive cells, and the CXCR3 and CXCR4 axes increase effector cell recruitment^22^. The interaction of interleukin 17 (IL-17) and interleukin 17 receptor (IL-17R) in the TME can regulate tumor growth and metastasis^23^. Tumor necrosis factor alpha (TNF-α) is an essential pro-inflammatory cytokine produced by tumors and can promote tumorigenesis^24^. Thus, the dynamic relationships between the robust DEGs and the TME in the occurrence of ESCC deserves further study.

Immune cells can recognize neoantigens produced by tumor cells with genomic alterations. Our CIBERSORT analysis indicated that changes in macrophages between tumors and normal tissues were particularly obvious. Tumor -associated macrophages (TAMs) are the most abundant inflammatory cell population in the TME. TAMs and their precursors compose the largest fraction of the myeloid infiltrate in most solid human malignancies^25^. Macrophages differentiate into M1 and M2 macrophages under different actions of the TME. M1 macrophages have a predominantly antitumour effect; in contrast, M2 macrophages secrete many immunoregulatory factors, such as cytokines, chemokines and metalloproteinases, which affect most aspects of ESCC progression by promoting tumor angiogenesis and lymphangiogenesis^26,27^. TAMs mainly perform M2-like functions. Our results showed that the infiltration levels of M0 and M1 macrophages in ESCC tissues were significantly higher than those in normal tissues. However, M2 macrophages were less abundant in ESCC tissues than in normal tissues. This “paradoxical” distribution of M2 macrophages may occur due to the high dynamics and heterogeneity of the TAM compartment. Our study presented only the approximate infiltration of immune cells in ESCC; more study is needed to investigate the diverse roles of immune cells in the TME.

We identified 9 hub genes among the robust DEGs by constructing a PPI network. Among the hub genes, CXCL1, IGFBP3, MMP3, MMP11, SERPINE1, SPP1 and VCAN exhibited upregulated expression in ESCC, while CDA exhibited downregulated expression. Because of the significant changes in macrophages in ESCC, we sought to determine whether the hub genes were associated with macrophages. Correlation analysis of immune cell infiltration with hub gene expression showed that the expression levels of CDA and CXCL1 were negatively associated with the macrophage infiltration level, while the expression levels of IGFBP3, MMP11, PLAU, SERPINE1, SPP1 and VCAN were positively associated with the macrophage infiltration level. CDA was downregulated in more than half of tumor cells and tissues, and DNA damage and genomic instability are consequences of CDA silencing. DNA methylation is a prevalent mechanism for the loss of CDA expression^28^. Zhang et al.^29^ showed that CXCL1 was highly expressed in cancer-associated fibroblasts (CAFs) and that CAF-secreted CXCL1 mediated radioresistance in ESCC by regulating DNA damage repair and the Mek/Erk signalling pathway. They also demonstrated that CAF-secreted CXCL1 was an independent prognostic factor for ESCC patients who received chemoradiotherapy. The cancer cell surface marker CD44 promotes tumor invasion and metastasis by mediating crosstalk between cancer cells and the TME. IGFBP3 mediates the induction of CD44-high cells by suppressing reactive oxygen species (ROS) in the esophageal cancer microenvironment^30^. Both MMPs and the uPA systems are serine proteases that mediate tumor progression through degradation of the ECM. High MMP11 expression has been found to be closely associated with poor prognosis in ESCC^31^, and circulating PLAU mRNA in peripheral blood can potentially serve as a biomarker of unfavourable prognosis in ESCC^32^. SERPINE1 has numerous pro-tumorigenic functions in sustaining proliferative signalling, resisting tumour cell death, and promoting angiogenesis, invasion, metastasis and cancer inflammation^33^. SPP1, a multifunctional ECM phosphoprotein secreted by several cell types, is involved in various biological functions, including wound healing, bone calcification, immune responses and tumor progression^34,35^. In addition, SPP1 has been reported to be involved in many aspects of head and neck cancer, lung cancer and gastric cancer^36–38^. A meta-analysis of 8 studies showed that SPP1 overexpression might serve as an excellent independent prognostic risk factor in 811 Chinese and Japanese ESCC patients^39^. VCAN, a CAF-related stromal protein, is an essential ECM component. Previous studies showed that VCAN was closely associated with the proliferation and metastasis of various types of tumor cells, such as gastric cancer, leukaemia and breast cancer cells^40–42^. In addition, a recent study showed that stromal expression of VCAN was strongly associated with worse overall and relapse-free survival in patients with ESCC^43^. The ROC curves showed that all 9 hub genes had relatively high diagnostic value for ESCC patients. The biological significance of intertumor and intratumor heterogeneity in ESCC has been discussed^44^. Moreover, the relationship between the molecular characteristics of tumor cells and the TME is extremely complex. Considering their important roles in the TME, the hub genes may have functions in immune escape. Exploration of these hub genes is expected to provide a new strategy for targeted TME therapy.

It is of crucial clinical significance to stratify patients with ESCC and construct a prognostic prediction model. Li et al*.*^45^ constructed an eight-lncRNA prognostic signature and nomogram based on the GEO and TCGA databases to improve the prediction of ESCC prognosis. Mao et al*.*^46^ found that a novel six-miRNA signature could be an independent biomarker for the survival prediction of ESCC patients. In our study, seven genes (IL18, PLAU, ANO1, SLCO1B3, CST1, NELL2 and MAGEA11) were used to construct a new Cox risk model that can predict the outcome of the high- and low-risk groups. The performance of this model classifier was verified with TCGA data. All AUC values for 3- and 5-year OS were greater than 0.65 in both the training and validation cohorts, indicating the good sensitivity and specificity of the prognostic signature for ESCC. We also revealed that the risk score could be used as a valuable independent prognostic indicator. The nomogram suggested that the 1-, 3-, and 5-year survival rates of patients with ESCC can be intuitively predicted based on the relative expression level of the 7 genes in our model.

In conclusion, our DEGs were derived from a greater amount of data (9 GEO datasets), and the screening criteria were more rigorous (|log2FC|> 2) than those used in previous studies. The distribution of infiltrating immune cells in ESCC was also analysed based on the 9 GEO datasets. The robust DEGs were found to participate in multiple processes in the TME. Clarifying the underlying mechanisms of the hub genes may improve the effect of targeted TME therapy. A Cox regression model was constructed based on the robust DEGs identified in the GSE53625 cohort, in which all patients with ESCC had undergone surgery. Thus, the risk model could be used to predict the prognosis of ESCC patients treated with esophagectomy.

## Methods

### Microarray data collection

Microarray datasets GSE17351, GSE20347, GSE29001, GSE38129, GSE45670, GSE53625, GSE70409, GSE75241 and GSE161533 were obtained from the Gene Expression Omnibus (GEO) database (https://www.ncbi.nlm.nih.gov/geo/). The basic information for the nine GEO datasets evaluated in the current study is provided in Table 1. Only esophageal squamous cell carcinoma (ESCC) tissue and normal tissue samples were selected from these datasets for further analysis. Each included dataset contained at least ten samples. The Cancer Genome Atlas (TCGA) RNA sequencing data (RPKM format) were downloaded from the UCSC Xena (https://tcga-xena-hub.s3.us-east-1.amazonaws.com/download/TCGA.ESCA.sampleMap%2FHiSeq.gz). In addition, clinical information was obtained from the GSE53625 dataset and the UCSC Xena.

### Differential expression analysis in ESCC

We used the “limma” package in R software (version 3.6.3, https://www.r-project.org/) to identify differentially expressed genes (DEGs) between ESCC tissues and normal tissues with the cutoff criteria |log2 fold change (FC)|> 2 and adjusted *P* < 0.05. After the upregulated and downregulated genes in each dataset were ranked by their FC values, we utilized the robust rank aggregation (RRA) algorithm to integrate the nine microarray datasets. Then, the “RobustRankAggreg” R package was used to identify the robust DEGs. Genes with |log2FC|> 2 and adjusted *P* < 0.05 were considered significant robust DEGs.

### Functional and pathway enrichment analyses

To determine the biological annotations of the robust DEGs identified as indicated above, Gene ontology (GO) functional enrichment and Kyoto Encyclopedia of Genes and Genomes (KEGG) pathway^47^ enrichment analyses were conducted using the “clusterProfiler” R package. The GO analysis included biological process (BP), cellular component (CC) and molecular function (MF) categories. An adjusted *P* < 0.05 was considered to indicate a statistically significant difference.

### Analysis of immune cell infiltration with the CIBERSORT algorithm

All gene expression matrices for each tissue sample were normalized and converted to matrices for 22 kinds of immune cells with the CIBERSORT algorithm (http://cibersort.stanford.edu/)^48^. The 22 kinds of immune cells included nine types of adaptive immune cells [memory B cells, naïve B cells, activated memory CD4^+^ T cells, resting memory CD4^+^ T cells, naïve CD4^+^ T cells, CD8^+^ T cells, follicular helper T cells, regulatory T cells (Tregs) and gamma delta T cells] and 13 types of innate immune cells [activated dendritic cells, resting dendritic cells, eosinophils, macrophages (M0–M2), activated mast cells, resting mast cells, monocytes, resting NK cells, activated NK cells, neutrophils and plasma cells]. R packages were used to evaluate the differences in the 22 immune cell subpopulations between ESCC and normal samples according to the filtering criterion *P* < 0.05. The discriminative value of the 22 immune cell populations in ESCC and normal tissues were visualized by principal component analysis (PCA)^49^.

### Identification of hub genes

The online Search Tool for the Retrieval of Interacting Genes (STRING) database (http://string-db.org/)^50^ was used to obtain the predicted interactions for the robust DEGs with medium confidence (> 0.4). The protein–protein interaction (PPI) network of the robust DEGs was visualized with Cytoscape software (Version 3.72, http://www.cytoscape.org/). The CytoHubba plugin in Cytoscape features 12 different algorithms to analyse PPI network topology: Maximal Clique Centrality (MCC), Density of Maximum Neighborhood Component (DMNC), Maximum Neighborhood Component (MNC), Degree, Component (EPC), BottleNeck, EcCentricity, Closeness, Radiality, Betweenness, Stress and ClusteringCoefficient^51^. The outputs of these algorithms can be integrated to identify hub genes.

### Analysis of hub genes

The differential expression of hub genes in ESCC was validated using the Gene Expression Profiling Interactive Analysis (GEPIA) database (http://gepia.cancer-pku.cn/)^52^. The normal samples in GEPIA include TCGA normal and Genotype Tissue Expression (GTEx) data (https://www.gtexportal.org/). Receiver operating characteristic (ROC) curves were utilized to assess the performance of the hub genes as biomarkers for distinguishing between cancer and normal tissues based on the GSE53625 dataset. The ROC curves were drawn and the area under the curve (AUC) values were calculated using GraphPad Prism 8.0 software (GraphPad Software, Inc., La Jolla, California). Correlations between hub genes and immune infiltration levels were assessed with Tumor Immune Estimation Resource (TIMER, https://cistrome.shinyapps.io/timer/)^53^.

### Construction and validation of the prognostic model

A total of 179 ESCC patients with reliable clinical prognostic information in the GSE53625 dataset were selected as the training group. After filtering out samples without overall survival (OS) data, 185 patients from the TCGA-ESCA dataset were selected as the validation cohort. Univariate Cox proportional hazards regression analysis was performed on the 152 robust DEGs to identify prognosis-related genes using the “survival” R package. Next, based on the above preliminarily identified significant genes, we constructed a multivariate Cox proportional hazards regression model and calculated risk scores for predicting the prognosis of ESCC patients. The risk score formula related to the prognostic signature was as follows: Risk score = Ʃ (*β*_*i*_ × *Exp*_*i*_), where *β*_*i*_ is the coefficient value, and *EXP*_*i*_ is the gene expression level. The ESCC patients were divided into the low-risk and high-risk groups based on the median risk score. A time-dependent ROC curve was generated with the “SurvivalROC” R package to assess the predictive power of the prognostic model.

### Independence analysis of the prognostic model and construction of the nomogram

Univariate and multivariate regression analyses were used to identify independent prognostic factors (including age, gender, grade, pathologic T stage, lymph node metastasis status, clinical stage and risk score) in patients with ESCC. The nomogram with calibration plots was constructed using the “rms” R package to predict the one-year, three-year and five-year survival probabilities. *P* < 0.05 was considered statistically significant.

## Supplementary Information

Supplementary Information.

## Data Availability

The datasets generated and/or analysed during the current study are available in the GEO repository (https://www.ncbi.nlm.nih.gov/geo/) and UCSC Xena(http://xena.ucsc.edu/).
