# Supplementary material for: Integrated bioinformatics analysis of differentially expressed genes and immune cell infiltration characteristics in Esophageal Squamous cell carcinoma
Source: Sci Rep. 2021 Aug 17;11:16696. doi: 10.1038/s41598-021-96274-y (PMC8371051; doi:10.1038/s41598-021-96274-y)
Supplement: Supplementary file 1 — Supplementary Information. [file 41598_2021_96274_MOESM1_ESM.pdf]

Integrated Bioinformatics Analysis of Differentially Expressed Genes and Immune Cell Infiltration Characteristics in Esophageal Squamous Cell Carcinoma

Zitong Feng[1,4], Jingge Qu[2], Xiao Liu[3,4], Jinghui Liang[1,4], Yongmeng Li[1,4], Jin Jiang[1,4], Huiying Zhang[1] and Hui Tian[1,*]

[1] Department of Thoracic Surgery, Qilu Hospital, Cheeloo College of Medicine, Shandong University, Jinan, Shandong, 250012, China

[2] Department of Rheumatology and Clinical Immunology, Peking Union Medical College Hospital, Peking Union Medical College & Chinese Academy of Medical Sciences, Beijing 100730, China.

[3] Department of Pulmonary and Critical Care Medicine, Qilu Hospital, Cheeloo College of Medicine, Shandong University, Jinan, Shandong, 250012, China

[4] Laboratory of Basic Medical Sciences, Qilu Hospital, Cheeloo College of Medicine, Shandong University, Jinan, Shandong, 250012, China

*Correspondence to Hui Tian, Department of Thoracic Surgery, Qilu Hospital, Cheeloo College of Medicine, Shandong University, Jinan, Shandong, 250012, China. Email: tianhuiql@126.com

Figure legends

Figure S1 Volcano plots of DEGs in (**A**) GSE17351, (**B**) GSE20347, (**C**) GSE29001, (**D**) GSE38129, (**E**) GSE45670, (**F**) GSE53625, (**G**) GSE70409, (**H**) GSE75241 and (**I**) GSE161533. The red and blue dots denote the upregulated and downregulated genes, respectively.

Figure S2 Hierarchical clustering heat maps of DEGs in GSE17351 (**A**), GSE20347 (**B**), GSE29001 (**C**), GSE38129 (**D**), GSE45670 (**E**), GSE53625 (**F**), GSE70409 (**G**), GSE75241 (**H**) and GSE161533 (**I**). The heatmaps were drawn using R software (version 3.6.3, https://www.r-project.org/).

Figure S3 Heat map visualizing the differences in immune cell infiltration between ESCC and normal tissues. The heatmap was drawn using R software (version 3.6.3, https://www.r-project.org/).

Figure S4 Nine hub genes were identified by intersecting the top 50 robust DEGs identified by the 12 algorithms in the cytoHubba plugin.

Figure S5 ROC curve analysis of the 9 hub genes. The AUCs of CDA (**A**), CXCL1 (**B**), IGFBP3 (**C**), MMP3 (**D**), MMP11 (**E**), PLAU (**F**), SERPINE1 (**G**), SPP1 (**H**) and VCAN (**I**) were 0.8816, 0.8303, 0.9627, 0.9462, 0.9975, 0.9822, 0.9344, 0.9890 and 0.9454, respectively.

Figure S6 Risk heat map constructed from the 7 robust DEGs based on 179 patients in GSE53625. The heatmap was drawn using R software (version 3.6.3, https://www.r-project.org/).

Table S1 The 152 robust DEGs in ESCC.

Name	P value	FDR	logFC
MMP1	4.46E-37	1.25E-32	6.121141
CRISP3	5.61E-32	1.57E-27	-5.98897
SPP1	1.79E-25	2.49E-21	4.097235
CLCA4	2.41E-24	2.25E-20	-3.85845
ENDOU	2.41E-24	2.25E-20	-4.04771
MMP10	3.72E-24	3.46E-20	3.738794
SCEL	4.57E-23	3.19E-19	-3.72889
LAMC2	2.24E-22	1.57E-18	2.685092
MAL	4.93E-22	2.75E-18	-4.07628
SPINK5	9.51E-22	4.43E-18	-3.58983
MMP12	1.53E-21	8.57E-18	4.072557
MMP3	6.50E-21	3.02E-17	3.871606
TMPRSS1	2.44E-20	9.72E-17	-3.2166
SLURP1	3.13E-20	1.09E-16	-3.38608
CRNN	4.40E-20	1.37E-16	-4.17321
HPGD	5.43E-20	1.52E-16	-3.48339
CXCL8	4.77E-20	1.90E-16	2.881366
PSCA	1.76E-19	4.48E-16	-2.99654
MMP13	1.96E-19	6.85E-16	3.165443
LAMB3	3.39E-19	1.05E-15	2.103863
TGM3	5.34E-19	1.24E-15	-3.46093
CRCT1	5.95E-19	1.28E-15	-3.54178
PLAU	4.67E-19	1.30E-15	2.345648
KLK13	7.86E-19	1.57E-15	-2.81834
GYS2	9.40E-19	1.75E-15	-3.47459
CLIC3	1.33E-18	2.32E-15	-3.19789
MAGEA6	1.12E-18	2.85E-15	3.501851
CEP55	2.67E-18	6.00E-15	2.120883
CST1	2.79E-18	6.00E-15	3.01805
CWH43	4.35E-18	7.15E-15	-2.88654
NELL2	4.12E-18	8.21E-15	2.469428
PTHLH	4.73E-18	8.81E-15	2.373483
ANO1	7.67E-18	1.26E-14	2.820893
RHCG	8.78E-18	1.36E-14	-2.66777
TPX2	9.22E-18	1.43E-14	2.116864
HOXC10	1.39E-17	2.04E-14	2.229939
CYP4B1	4.26E-17	6.26E-14	-3.4042
TFAP2B	6.36E-17	8.88E-14	-2.86623
KRT4	7.26E-17	9.66E-14	-3.00055

UPK1A	8.47E-17	1.07E-13	-2.7937
ANXA9	9.36E-17	1.14E-13	-2.44153
SCNN1B	1.26E-16	1.46E-13	-2.58426
APOBEC3	1.25E-16	1.66E-13	2.57927
CXCL10	1.38E-16	1.76E-13	2.239305
SERPINH1	1.67E-16	1.94E-13	2.277872
ODC1	2.18E-16	2.35E-13	2.017337
CXCL1	2.39E-16	2.47E-13	2.431829
IFI6	3.40E-16	3.16E-13	2.100108
ECM1	2.96E-16	3.31E-13	-2.73214
SERPINE1	3.94E-16	3.55E-13	2.012127
CEACAM7	3.86E-16	4.14E-13	-2.56553
VCAN	5.11E-16	4.38E-13	2.045332
SNX10	6.60E-16	5.42E-13	2.138982
MMP9	7.06E-16	5.48E-13	2.206237
EPCAM	7.58E-16	5.63E-13	2.018783
COL10A1	7.66E-16	5.63E-13	2.890866
INHBA	8.23E-16	5.89E-13	2.437083
IGFBP3	9.39E-16	6.29E-13	2.07048
POSTN	9.46E-16	6.29E-13	2.258127
CH25H	7.14E-16	7.26E-13	-2.11598
TGM1	7.34E-16	7.26E-13	-2.42209
MGLL	7.63E-16	7.26E-13	-2.44186
GDPD3	7.80E-16	7.26E-13	-2.52199
MFAP2	1.20E-15	7.77E-13	2.421684
ECT2	1.33E-15	8.08E-13	2.061104
HLF	1.16E-15	1.04E-12	-2.16377
FLG	1.26E-15	1.10E-12	-3.15933
TGFBI	1.95E-15	1.12E-12	2.333372
COL1A2	1.97E-15	1.12E-12	2.113835
HOXD11	2.39E-15	1.24E-12	2.412571
UCHL1	2.39E-15	1.24E-12	2.001604
ISG15	3.21E-15	1.51E-12	2.190059
PPP1R3C	1.83E-15	1.55E-12	-2.76808
CEACAM5	2.31E-15	1.90E-12	-2.35333
ZNF185	2.56E-15	2.04E-12	-2.09441
KAT2B	2.82E-15	2.17E-12	-2.2741
SLC6A1	2.88E-15	2.17E-12	-2.13637
ADH1B	3.42E-15	2.45E-12	-3.13233
IL18	3.42E-15	2.45E-12	-2.50885
EPS8L1	3.65E-15	2.55E-12	-2.10115
GPD1L	3.89E-15	2.60E-12	-2.0124

SERPINB2	3.90E-15	2.60E-12	-2.58002
KIF14	6.32E-15	2.63E-12	2.074675
HOXB7	6.46E-15	2.63E-12	2.084526
SLCO1B3	6.51E-15	2.63E-12	2.019433
CEACAM6	4.40E-15	2.86E-12	-2.33395
SYNPO2L	4.68E-15	2.97E-12	-2.68464
HOXD10	8.68E-15	3.37E-12	2.808399
SULT2B1	6.70E-15	4.16E-12	-2.11791
CXCR2	7.31E-15	4.44E-12	-2.76407
PPL	7.97E-15	4.74E-12	-2.24933
COL11A1	1.66E-14	5.87E-12	2.828742
IL1RN	1.21E-14	6.90E-12	-2.01716
PCP4	1.21E-14	6.90E-12	-2.62905
PLA2G7	2.20E-14	7.31E-12	2.240873
C1orf116	1.39E-14	7.59E-12	-2.12357
SIM2	1.50E-14	8.06E-12	-2.13479
CYP2C18	1.69E-14	8.76E-12	-2.22709
GABRP	1.76E-14	8.92E-12	-2.46541
EDN3	2.16E-14	1.08E-11	-2.59652
EMP1	2.77E-14	1.33E-11	-2.49779
CEACAM1	3.12E-14	1.48E-11	-2.01058
MMP11	5.94E-14	1.77E-11	2.586406
SASH1	3.95E-14	1.77E-11	-2.18282
KLK11	3.96E-14	1.77E-11	-2.00804
PRSS3	4.54E-14	1.98E-11	-2.0192
ZNF365	7.13E-14	3.06E-11	-2.20171
COL1A1	1.27E-13	3.47E-11	2.052622
GALNT12	9.43E-14	3.92E-11	-2.17453
SERPINB3	1.16E-13	4.69E-11	-2.10038
PTK6	1.32E-13	5.18E-11	-2.12743
ABLIM3	1.39E-13	5.39E-11	-2.21291
HOPX	1.53E-13	5.79E-11	-2.31901
TMPRSS1	1.65E-13	6.06E-11	-2.79043
NUCB2	1.83E-13	6.55E-11	-2.03552
FCER1A	2.08E-13	7.36E-11	-2.46398
SPINK7	2.13E-13	7.44E-11	-2.64113
CFD	3.57E-13	1.21E-10	-2.23364
BBOX1	3.85E-13	1.29E-10	-2.46494
C7	4.33E-13	1.42E-10	-2.54896
FMO2	4.80E-13	1.52E-10	-2.42543
ABCA8	5.47E-13	1.72E-10	-2.38633
MYH11	6.29E-13	1.93E-10	-2.28681

COL5A2	9.54E-13	2.08E-10	2.005395
CDA	6.94E-13	2.08E-10	-2.05433
BLNK	7.65E-13	2.25E-10	-2.18081
MAGEA4	1.89E-12	3.84E-10	2.228391
CNN1	1.49E-12	4.00E-10	-2.22058
ACPP	1.69E-12	4.49E-10	-2.10769
CRISP2	1.73E-12	4.55E-10	-2.04714
CXCL6	2.60E-12	4.94E-10	2.094242
SLC16A6	2.10E-12	5.41E-10	-2.07378
SERPINB1	2.33E-12	5.86E-10	-2.05516
GPX3	2.87E-12	6.79E-10	-2.34909
CXCL11	4.57E-12	7.83E-10	2.017483
TTC9	3.50E-12	8.05E-10	-2.02476
ALOX12	4.05E-12	9.11E-10	-2.1754
PLN	4.81E-12	1.05E-09	-2.09455
FAM3D	6.00E-12	1.24E-09	-2.19212
EPB41L3	7.18E-12	1.45E-09	-2.1977
ATP1A2	1.55E-11	2.75E-09	-2.49221
SPRR3	2.06E-11	3.47E-09	-2.24895
CTHRC1	3.86E-11	4.99E-09	2.199487
CASQ2	4.34E-11	6.77E-09	-2.19462
PADI1	5.97E-11	8.97E-09	-2.17501
PRSS27	1.04E-10	1.43E-08	-2.05433
OGN	1.34E-10	1.74E-08	-2.26316
KRT78	3.62E-10	4.12E-08	-2.34254
SH3BGRL2	4.38E-10	4.78E-08	-2.12336
S100A7	7.74E-10	6.59E-08	2.084753
CHRDL1	2.63E-09	2.10E-07	-2.05078
SFTA2	2.37E-07	9.71E-06	-2.02615

Figure S1

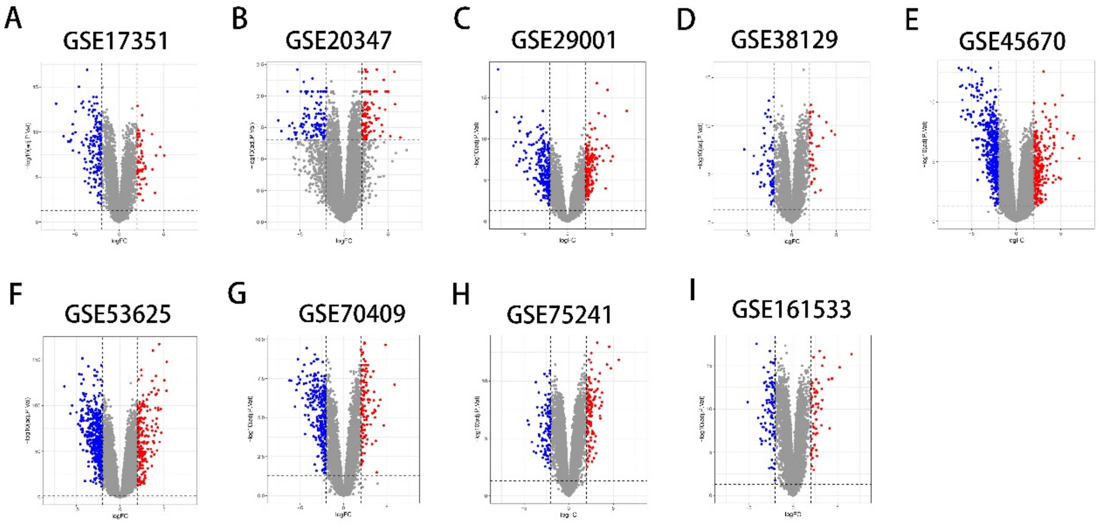
A
GSE17351
B
GSE20347
C
GSE29001
D
GSE38129
E
GSE45670
F
GSE53625
G
GSE70409
H
GSE75241
I
GSE161533

Figure S2

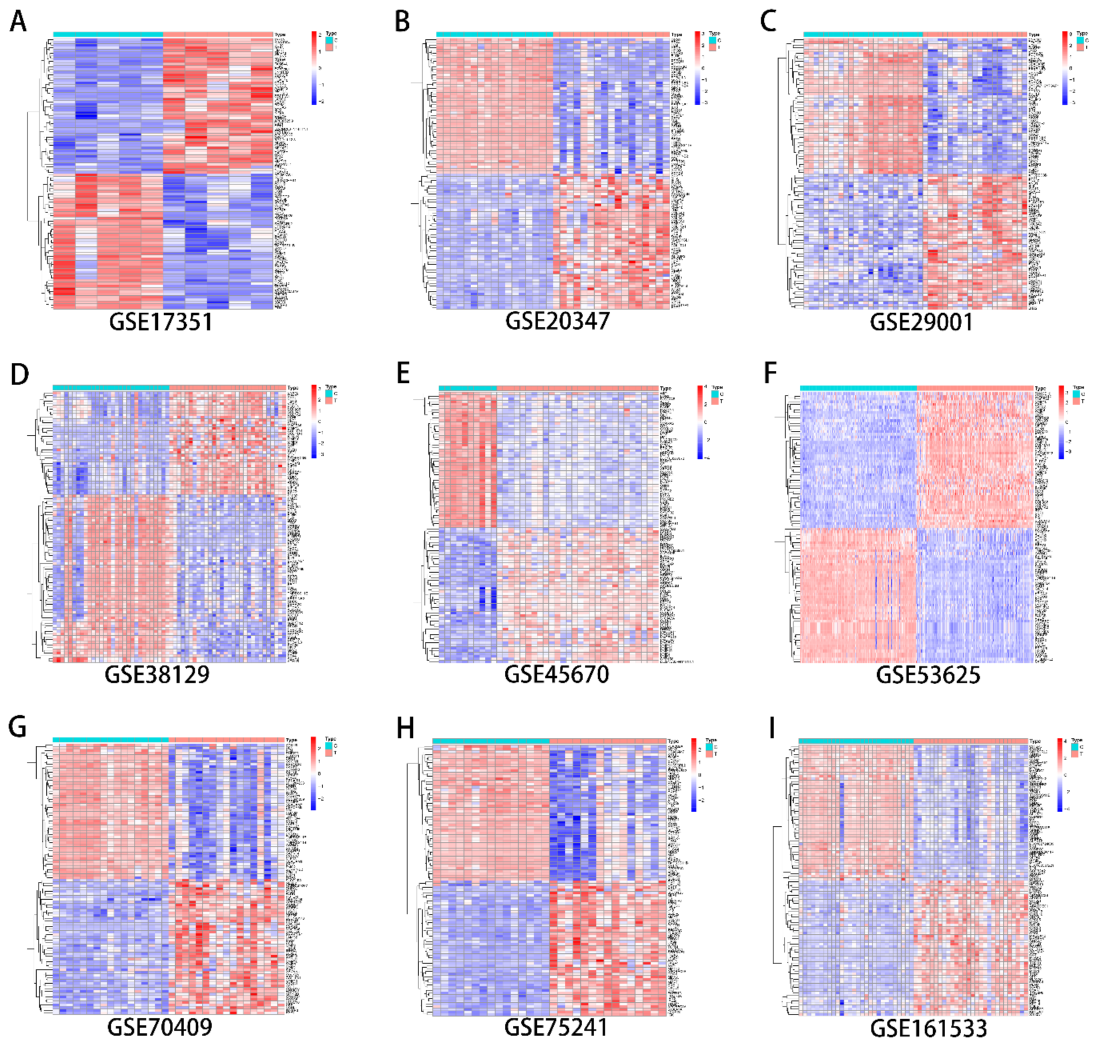
A
GSE17351
B
GSE20347
C
GSE29001
D
GSE38129
E
GSE45670
F
GSE53625
G
GSE70409
H
GSE75241
I
GSE161533

Figure S3

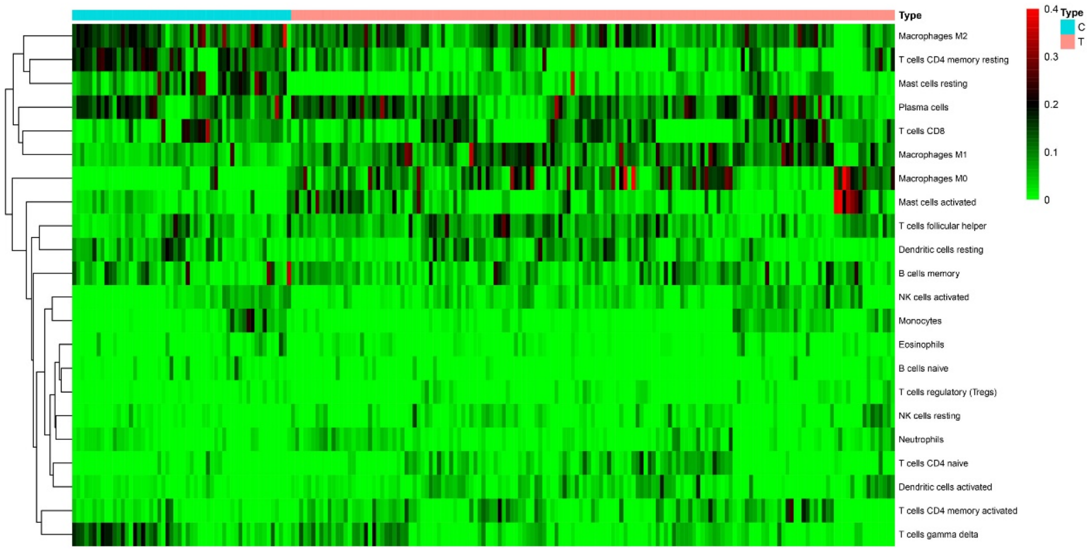
Type
Macrophages M2
T cells CD4 memory resting
Mast cells resting
Plasma cells
T cells CD8
Macrophages M1
Macrophages M0
Mast cells activated
T cells follicular helper
Dendritic cells resting
B cells memory
NK cells activated
Monocytes
Eosinophils
B cells naive
T cells regulatory (Tregs)
NK cells resting
Neutrophils
T cells CD4 naive
Dendritic cells activated
T cells CD4 memory activated
T cells gamma delta
0.4
0.3
0.2
0.1
0
Type
C
T

Figure S4

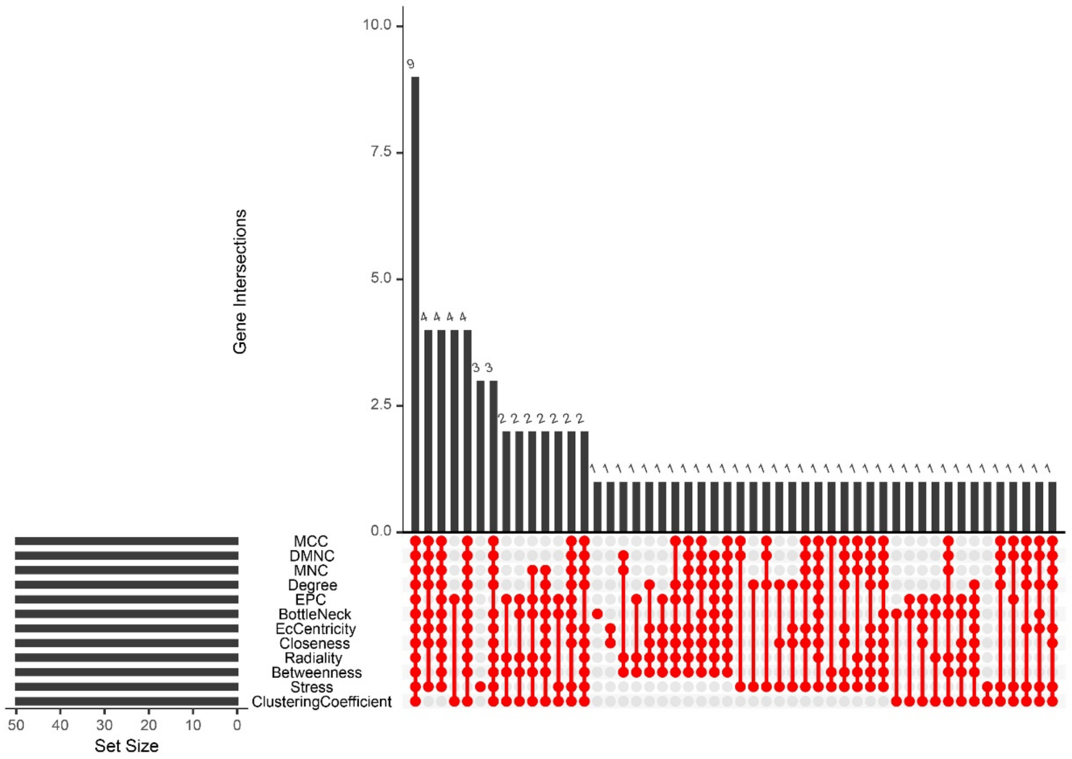

Gene Intersections
10.0
7.5
5.0
2.5
0.0
9
4 4 4 4
3 3
2 2 2 2 2 2 2
MCC
DMNC
MNC
Degree
EPC
BottleNeck
EcCentricity
Closeness
Radiality
Betweenness
Stress
ClusteringCoefficient
50 40 30 20 10 0
Set Size

Figure S5

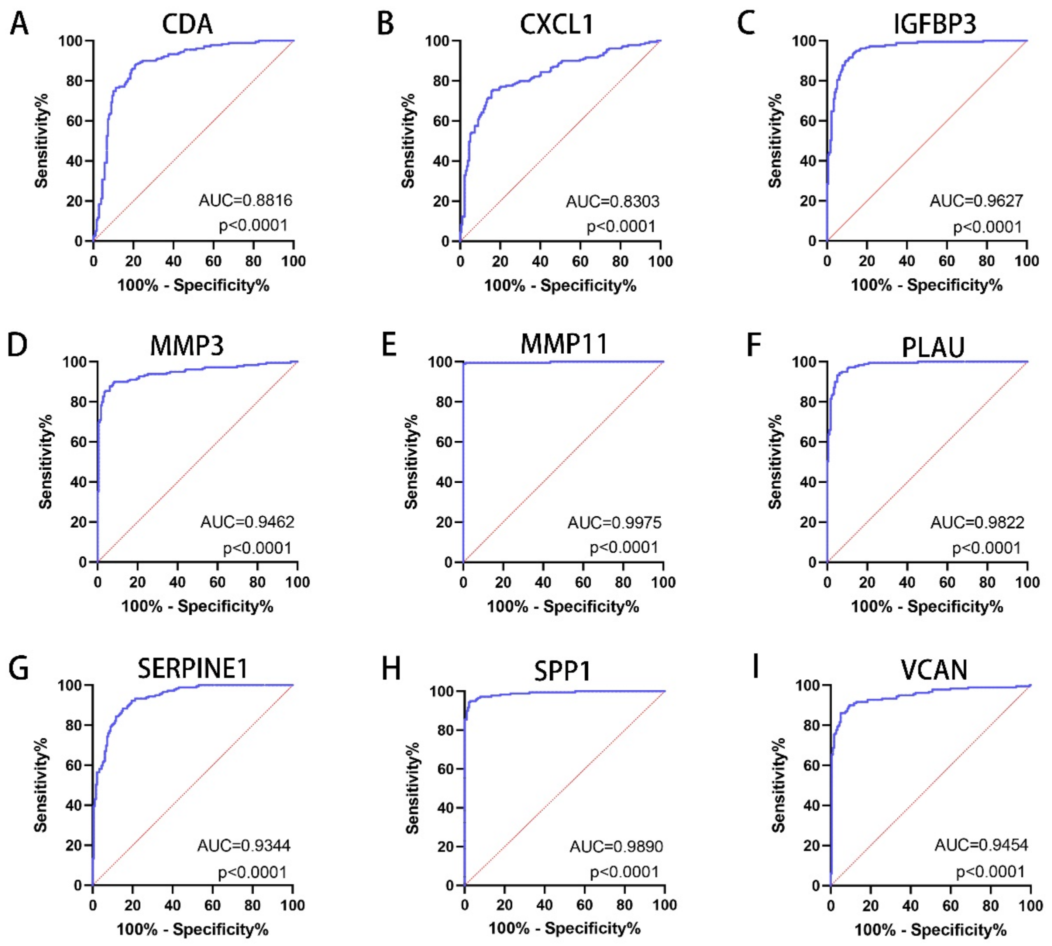
A
CDA
AUC=0.8816
p<0.0001
B
CXCL1
AUC=0.8303
p<0.0001
C
IGFBP3
AUC=0.9627
p<0.0001
D
MMP3
AUC=0.9462
p<0.0001
E
MMP11
AUC=0.9975
p<0.0001
F
PLAU
AUC=0.9822
p<0.0001
G
SERPINE1
AUC=0.9344
p<0.0001
H
SPP1
AUC=0.9890
p<0.0001
I
VCAN
AUC=0.9454
p<0.0001
Sensitivity%
100% - Specificity%

Figure S6

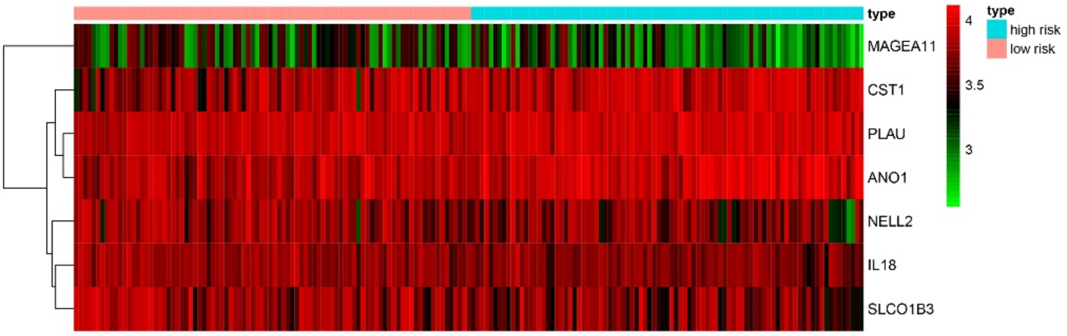
type
MAGEA11
CST1
PLAU
ANO1
NELL2
IL18
SLCO1B3
4
3.5
3
type
high risk
low risk
